# In silico identification and biological evaluation of a selective MAP4K4 inhibitor against pancreatic cancer

**DOI:** 10.1080/14756366.2023.2166039

**Published:** 2023-01-22

**Authors:** Chao-Di Chang, Min-Wu Chao, Hsueh-Yun Lee, Yi-Ting Liu, Huang-Ju Tu, Ssu-Ting Lien, Tony Eight Lin, Tzu-Ying Sung, Shih-Chung Yen, Sing-Han Huang, Kai-Cheng Hsu, Shiow-Lin Pan

**Affiliations:** a Ph.D. Program in Drug Discovery and Development Industry, College of Pharmacy, Taipei Medical University, Taipei, Taiwan; b School of Medicine, College of Medicine, National Sun Yat-sen University, Kaohsiung, Taiwan; c Institute of Biopharmaceutical Sciences, College of Medicine, National Sun Yat-sen University, Kaohsiung, Taiwan; d School of Pharmacy, College of Pharmacy, Taipei Medical University, Taipei, Taiwan; e TMU Research Center of Cancer Translational Medicine, Taipei Medical University, Taipei, Taiwan; f Graduate Institute of Cancer Biology and Drug Discovery, College of Medical Science and Technology, Taipei Medical University, Taipei, Taiwan; g Ph.D. Program for Cancer Molecular Biology and Drug Discovery, College of Medical Science and Technology, Taipei Medical University, Taipei, Taiwan; h Biomedical Translation Research Center, Academia Sinica, Taipei, Taiwan; i Warshel Institute for Computational Biology, The Chinese University of Hong Kong (Shenzhen), Shenzhen, Guangdong, People’s Republic of China; j Graphen Inc, New York, NY, USA; k TMU Research Center for Drug Discovery, Taipei Medical University, Taipei, Taiwan; l Cancer Center, Wan Fang Hospital, Taipei Medical University, Taipei, Taiwan

**Keywords:** MAP4K4, JNK signalling pathway, pancreatic cancer, structure-based virtual screening, kinase inhibitor

## Abstract

Inhibiting a specific target in cancer cells and reducing unwanted side effects has become a promising strategy in pancreatic cancer treatment. MAP4K4 is associated with pancreatic cancer development and correlates with poor clinical outcomes. By phosphorylating MKK4, proteins associated with cell apoptosis and survival are translated. Therefore, inhibiting MAP4K4 activity in pancreatic tumours is a new therapeutic strategy. Herein, we performed a structure-based virtual screening to identify MAP4K4 inhibitors and discovered the compound F389-0746 with a potent inhibition (IC_50_ 120.7 nM). The results of kinase profiling revealed that F389-0746 was highly selective to MAP4K4 and less likely to cause side effects. Results of in vitro experiments showed that F389-0746 significantly suppressed cancer cell growth and viability. Results of in vivo experiments showed that F389-0746 displayed comparable tumour growth inhibition with the group treated with gemcitabine. These findings suggest that F389-0746 has promising potential to be further developed as a novel pancreatic cancer treatment.

## Graphical abstract

**Figure:**
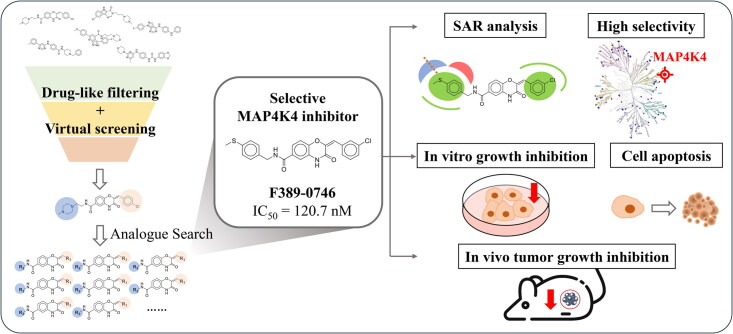

**Highlights**
- We identified a novel MAP4K4 inhibitor using a structure-based virtual screening strategy.
- Kinase profiling showed high selectivity towards MAP4K4.
- The identified inhibitor suppressed pancreatic cancer cell growth and viability in vitro.
- The inhibitor downregulated the MKK4-JNK signalling pathway.
- The inhibitor suppressed tumour growth in vivo.

## Introduction

Pancreatic cancer is the fourth leading cause of cancer death and has a 5-year survival rate of ≤15% because of the lack of appropriate diagnosis strategies and treatments^1^. Early pancreatic tumours are located deep inside the body, which is difficult to detect during routine exams^2^. Surgery and chemotherapy are the main treatments for pancreatic cancer. However, the treatments display a low success rate in removing the tumour at late stages. Surgery removes the entire tumour tissue at the primary site for metastasis prevention. Unfortunately, less than 20% of pancreatic cancer patients are surgically resectable^3^. Chemotherapy can be used at all pancreatic cancer stages to limit cancer cell growth by damaging the DNA and interfering with cell replication. The most commonly used chemotherapeutic drug for pancreatic cancer treatment is gemcitabine, a nucleoside analogue designed to inhibit DNA synthesis and affect the growth of cancer cells ^4^. Nevertheless, chemotherapies usually cause unpleasant side effects and are ineffective at advanced stages because the dysplastic stroma interferes with drug delivery^5^^,^^6^. In comparison, targeted therapy is a promising strategy to improve clinical outcomes by inhibiting specific targets in pancreatic cancer cells and may have fewer side effects^6^.

Mitogen-activated protein kinase kinase kinase kinase 4 (MAP4K4) is a member of the serine/threonine-protein kinase superfamily. The MAP4K4 protein structure contains an N-terminal kinase domain, coiled-coil domain, C-terminal hydrophobic leucine-rich citron homology (CNH) domain, and interdomain that connects the kinase domain and CNH domain^7^. Phosphorylation on the N-terminal kinase domain of MAP4K4 is the key to activating the function of MAP4K4 in the human body^7^. MAP4K4 is expressed in the human brain and liver. MAP4K4 phosphorylates the substrate, moesin, to enable membrane extension and retraction during the migration of epithelial cells^8^. Some studies reveal that MAP4K4 is associated with several malignancies, such as systemic inflammation^9^, lung tissue inflammation^9^, and cancer^10–14^. In pancreatic cancer, nearly half of patients were found to overexpress MAP4K4 in the pancreas compared to healthy individuals^15^. Clinicopathologic evidence also suggests that MAP4K4 overexpression in pancreatic cancer strongly correlates with poor overall survival and recurrence-free survival. Tumour size and metastasis are also associated with MAP4K4 expression^15^. A review study suggests that the signalling pathways regulated by MAP4K4 are responsible for cancer progression^10^. Therefore, inhibiting MAP4K4 and the downstream signalling pathways is the key to inhibiting tumour growth.

MAP4K4 is involved in the regulation of JNK, NF-κB, and Notch signalling pathways to promote cancer cell growth, cell cycle arrest, and apoptosis in different cancer types^14^^,^^16^^,^^17^. In the progression of gastric cancer and NSCLC, MAP4K4 was found to promote cancer cell growth, apoptosis, and migration by regulating Notch and NF-κB signalling pathways^16^. In recent years, mounting evidence has revealed that MAP4K4 manipulates the activity of the JNK signalling pathway to promote the proliferation, invasion, and metastasis of pancreatic cancer cells^14^^,^^18^. Through mitogen-activated protein kinase kinase 4 (MKK4), MAP4K4 manipulates the JNK signalling pathway in response to TNF-α activation in pancreatic cancer progression^14^^,^^17^^,^^19^. Positive feedback regulation of the MKK4-JNK signalling pathway is the key to promoting pancreatic cancer^18^.

MAP4K4 regulates biological behaviours in many malignancies by phosphorylating MKK4. Phosphorylation of MKK4 on S257 and T261 by MAP4K4 would further enhance the JNK phosphorylation^20^. Phosphorylated MKK4 directly phosphorylates JNK on T183 and Y185 to activate the JNK signalling pathway^21^^,^^22^. JNK has a common substrate site at the C-terminus and a glutamate aspartate domain at the N-terminus that interacts with MKK4^23^. JNK can phosphorylate c-Jun, which then forms a heterodimer with c-Fos, known as the activator protein-1 (AP-1) transcription factor^24^. The AP-1 transcription factor then enters the cell nucleus to translate proteins related to cell apoptosis, cell survival, the proliferation of mitogens (e.g., MMPs and VEGF), and system inflammation^22^^,^^25^^,^^26^. Recent studies indicated that JNK signalling pathway regulates cell survival in response to proinflammatory cytokines and growth factors, which are essential in cancer development^25^^,^^27^. In summary, MAP4K4 and its role in regulating the JNK signalling pathway mediate many proliferation-related functions to promote cancer development. Therefore, identifying novel MAP4K4 inhibitors with potency and high selectivity is important to suppress JNK-induced proliferation and cell growth.

Currently, there are four MAP4K4 inhibitors available: PF-06260933, MAP4K4-IN-3, GNE-220, and GNE-495. PF-06260933 and MAP4K4-IN-3 were directly derived from a potent MAP4K4 inhibitor identified from an internal Pfizer file^28^. The aminopyridine moiety of PF-06260933 was designed to interact with the surrounding residues and generate a hydrogen bond with D115. MAP4K4-IN-3 has improved overall drug-like properties and is more suitable for developing into a lead compound than PF-06260933^28^. GNE-220 and GNE-495 were designed using a structure-based method^29^. GNE-495 has a better potency due to an amide linker moiety, which forms a hydrogen bond with the residue C108^29^. Moreover, GNE-495 has better potency than GNE-220. These four MAP4K4 inhibitors are currently in the preclinical stages.

In this study, we used a structure-based virtual screening (SBVS) strategy to identify potential MAP4K4 inhibitors. Following the validation by kinase activity assay, a novel MAP4K4 inhibitor was identified. A structure-activity relationship (SAR) analysis was then performed to study the binding mechanism between the inhibitor and its analogues. In addition, in vitro cell-based assays and an in vivo xenograft model were used to investigate the effects of the MAP4K4 inhibitor in pancreatic cancer.

## Materials and methods

### Compounds and molecular docking

Compounds were obtained from the ChemDiv database (https://www.chemdiv.com/) for virtual screening. Compounds were then filtered by drug-like properties. The quantitative estimate of drug-likeness (QED), which provides a quantitative method for determining the drug-likeness properties of each compound, was calculated^30^. In this study, compounds with QED score of <0.25 were removed. Lipinski and Veber’s rules were then applied to select compounds more likely to be absorbed, distributed, metabolised, and excreted by the human body^31–33^. Potential pan-assay interference compounds (PAINS) were further removed because these compounds are often false positives^34^. The remaining compounds were then docked into the binding site of MAP4K4 using a molecular docking program, LeadIT^35^. LeadIT was designed based on an interaction model LUDI^36^, which calculates interactions between a compound and residues of a binding site. Docking poses and score of compounds were generated by LeadIT. All docking parameters used the default settings. The crystal structure of MAP4K4 (PDB ID: 4OBP) was downloaded from the RCSB Protein Data Bank^37^. Water molecules in the protein structure were removed. The binding site was determined to be 12 Å from the co-crystal ligand (HET ID: 2QU). Compounds were protonated in an aqueous solution and docked to the binding site using the default settings.

### Kinase inhibition measurement

The Z’LYTE assay (ThermoFisher Scientific) was used to measure the efficacy of potential inhibitors (https://www.thermofisher.com/selectscreen). Test compounds were dissolved in 1% DMSO and diluted to a 2X working concentration with a kinase mixture. Then, the solution, ATP solution, and all reagents were mixed and incubated for one hour. Finally, a fluorescence plate and ELISA reader were used to measure the results.

### Molecular field map

A SAR analysis was conducted using Forge, which identifies molecular force fields of activity cliffs for important regions essential to compound activity^38^. The molecular force fields were generated to examine the critical regions of compounds and included types of positive and negative electrostatic or hydrophobic potential. Forge generated a 3D map that displayed the activity cliffs by comparing differences in the molecular force fields and potencies between compounds. The activity cliffs represented favourable and unfavourable electrostatic and hydrophobic sites essential to potency. In this study, a structure comparison between analogues was used to analyse how a difference in the functional group impacts potency. All molecular force fields and the 3D map were generated using the default settings.

### Cell culture

Two human pancreatic cancer cell lines obtained from the Bioresource Collection and Research Center (BCRC) (https://catalog.bcrc.firdi.org.tw/) were used in this study, including Panc-1 and AsPC-1. The human pancreatic cancer cell line, Panc-1, was maintained in the DMEM medium supplement with 10% FBS. Nutritional components, including L-glutamine, sodium bicarbonate, and glucose, were added to the medium following the suggestion from BCRC. The human pancreatic cancer cell line, AsPC-1, was maintained in the RPMI-1640 medium supplement with 10% FBS. Nutritional components, including L-glutamine, sodium bicarbonate, glucose, HEPES, and sodium pyruvate were added to the medium following the suggestion from BCRC. Both cell lines were incubated at 37 °C in a 5% CO_2_ incubator.

### Cell proliferation assay and cell viability assay

A sulforhodamine B (SRB) assay was used to measure the antiproliferative effect of a compound. The SRB assay was performed using a standard method as described previously^39^. Two pancreatic cancer cell lines were seeded in 96-well plates at a density of 5,000 cells per well overnight. The next day, 10% of trichloroacetic acid (TCA) was added to the basal group to maintain the cells at the initial density. After treating with different concentrations of a compound dissolved in 0.1% dimethyl sulfoxide (DMSO) for 72 and 96 h, cells were fixed with TCA for an additional 15 min and stained with 0.4% SRB in 1% acetic acid for 5 min. Then, the cells were washed three times with 1% acetic acid after 5–10 min of SRB staining to wash out unbound SRB crystals. The cells were dissolved in a 10 mM Tris base solution. At last, the absorbance at 515 nm was measured using a microplate reader. The absorbance of each well was normalised with the basal group, which was treated with DMSO. The concentration that led to 50% cell growth inhibition was determined using non-linear regression analysis (GI_50_, 50% growth inhibition).

For cell viability measurement, a 3–(4,5-dimethylthiazol-2-yl)-2,5-diphenyltetrazolium bromide (MTT) assay was used to measure cell metabolic activity. The MTT assay was performed using a standard method as described previously^40^. Briefly, pancreatic cancer cells were seeded at 5,000 cells per well in 96-well plates and incubated at 37 °C. Then, test compounds dissolved in DMSO were administered to the cells at different concentrations for 72 or 96 h. Next, 0.5 mg/mL of the MTT solution dissolved in the respective culture mediums was prepared. The MTT solution was added to the plates and stained for one hour in a 37 °C in a 5% CO_2_ incubator. The remaining MTT solution was suctioned. Next, cells were pelleted and lysed with 100 μL DMSO, and absorbance was measured at 550 nm using a microplate reader. The absorbance of each well was normalised with the control group treated with DMSO. The concentration that caused 50% cell viability inhibition was determined using non-linear regression analysis.

### Cell cycle analysis

The cell cycle was determined by flow cytometry, and the detailed procedures were shown as previously described^41^. Briefly, pancreatic cancer cells were trypsinized and seeded in 6 cm culture dishes (200,000 cells/dish) one day before the treatment. Then, the cells were treated with 0.1% DMSO (vehicle) or 3, 10, and 30 µM compounds for 24, 48, and 72 h. The cells were trypsinized and fixed overnight with ice-cold 75% (v/v) ethanol at −20 °C. Then, the cells were centrifuged and washed using phosphate-buffered saline (PBS). The cells were resuspended in DNA extraction buffer (0.2 M Na_2_HPO_4_, 0.1 M citric acid, pH 7.8) for at least one hour. Next, the cells were stained with propidium iodide (PI)-containing staining buffer (PI 80 mg/ml, 0.1% Triton X-100, 100 mg/m RNase A in PBS). The DNA content of single cells was measured with a BD Accuri^TM^ C6 flow cytometer.

### Western blot analysis

Pancreatic cancer cell lines were treated with a compound at concentrations of 3, 10, and 30 μM for 24, 48, and 72 h. Cells were lysed with sodium dodecyl sulfate (SDS)-lysis buffer (1% SDS, ten mM Tris HCl buffer, and 2 mM EDTA) for 30 min. Cell lysates were sonicated for 10 s per sample and were centrifuged at 14,000 rpm at 4 °C for 15 min. The protein concentration in the cell lysate was quantified using the Thermo Scientific Pierce™ BCA Protein Assay Kit. 20 mg of protein was loaded into SDS-PAGE and separated with a 130-V current for two hours. Then, the proteins were transferred from an SDS-PAGE to a polyvinylidene difluoride (PVDF) membrane with a 400-mA current for two hours. The membranes were slowly shaken in 10% non-fat milk dissolved in 1X Tris-Buffered Saline and 0.1% Tween 20 detergent (TBST) for one hour at room temperature for blocking. After washing three times with TBST, the membranes were incubated with the specific primary antibodies at 4 °C overnight. The secondary antibodies were dissolved in 5% non-fat milk containing TBST in a ratio of antibody versus TBST equal to 1:5000. Finally, the signal was detected with enhanced chemiluminescence (ECL).

### In vivo animal model

The in vivo animal experiment was performed using a xenograft model, which followed ethical standards. The protocol of the in vivo experiment was reviewed and approved by the Animal Use and Management Committee of Taipei Medical University (IACUC approved No. LAC-2020-0455).

For the in vivo xenograft model, four-week-old male nude mice were injected subcutaneously with Panc-1 cells at a cell density of 10^7^ cells/mouse. To facilitate tumour growth, the same volume of Corning^®^ Matrigel^®^ Basement Membrane Matrix High Concentration (HC) was also injected into the right flank of each animal. When the tumour volume grew up to around 100 mm^3^, the mice were divided evenly into three groups: the control group, which received the vehicle (1% carboxymethyl cellulose, CMC and 0.5% Tween 80) orally daily; the positive control group, which received gemcitabine at 100 mg/kg once a week; the treatment group, which received the identified inhibitor at 200 mg/kg daily. The inhibitor was dissolved in the vehicle, and gemcitabine was dissolved in 0.5% Cremophor EL, 0.5% DMSO, and 90% dextrose. Tumour volume (*V*) was measured twice a week by the following equation: *V* = l*w2/2, where *w* and *l* are the width and length of the tumour, respectively.

### Analysis of MAP4K4 gene expression in pancreatic cancer

The gene expression RNAseq dataset (TPM) of pancreatic cancer (PAAD) was obtained from the UCSC Xena project^42^, which contains 332 normal and 179 tumour samples. Fold change (FC) and the *p* values were calculated to compare the MAP4K4 gene expression level between normal and tumour samples using the limma package^43^. PAAD RNA-Seq data with complete survival status from The Cancer Genome Atlas (TCGA) databases^44^ was used to analyse the relationship between prognostic significance and MAP4K4 expression. Overall survival (OS) represented the time interval from the date of diagnosis to death. The OS was censored at the last follow-up for living patients. Here, the quartiles of the RNA-Seq expression data were utilised to divide patients into two groups, including high-risk (75th percentile; *n* = 45) and low-risk (25th percentile; *n* = 45) patients. Finally, the Kaplan-Meier analysis of their association with survival was performed for comparison. The Kaplan-Meier analysis and log-rank test analysis were performed using the R TCGAbiolinks (v. 2.21.1) package^45^.

## Results

### Study overview

**Figure 1.**
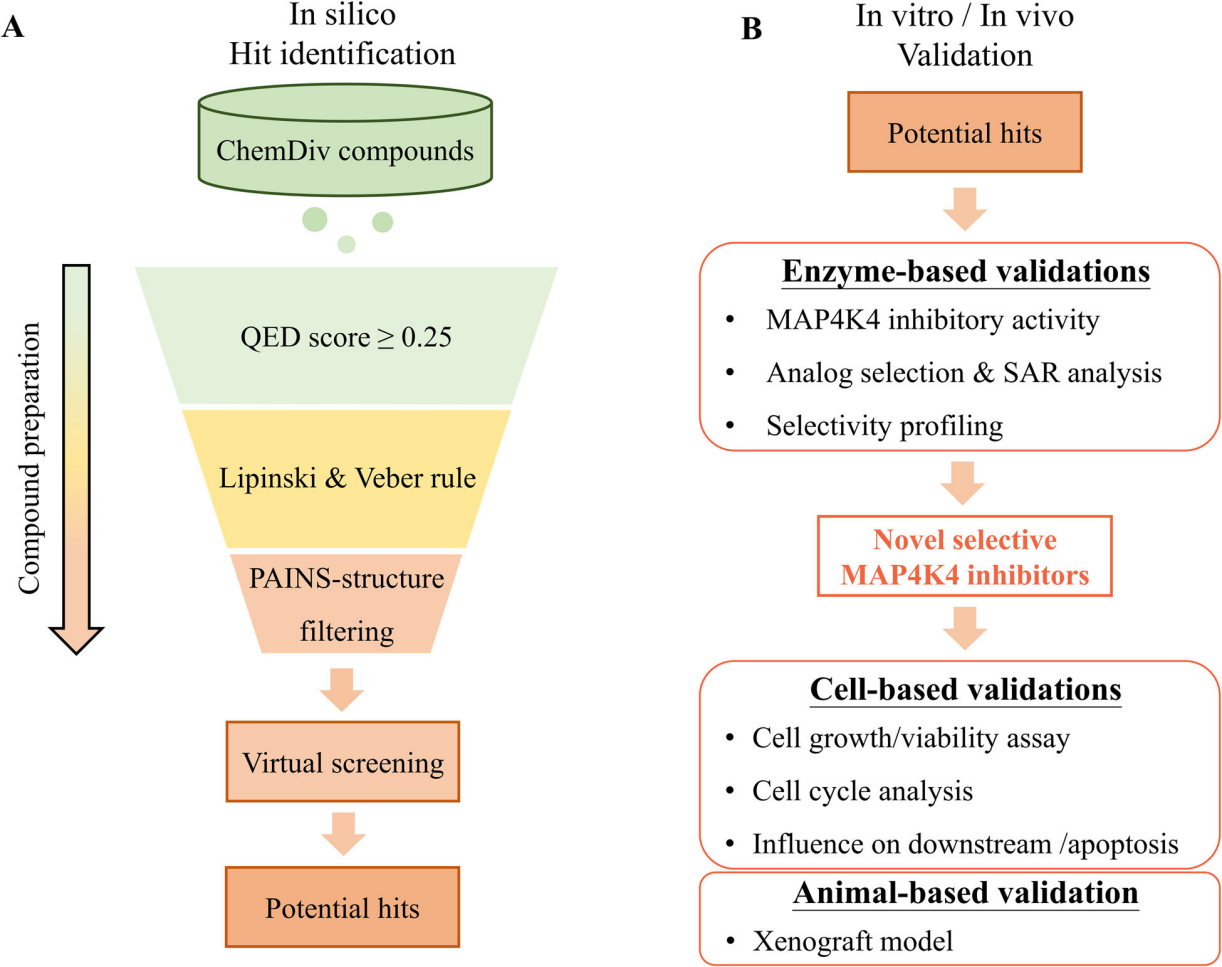
Workflow of identifying novel MAP4K4 inhibitors. (A) Steps of screening strategy. (B) In vitro and in vivo validation of potential MAP4K4 inhibitors.

In this study, an SBVS strategy was used to identify potential MAP4K4 inhibitors. Then, in vitro and in vivo experiments were performed to evaluate the effect of the inhibitor (Figure 1). Firstly, compounds were obtained from the ChemDiv database and filtered by drug-like properties. The remaining compounds were docked to the MAP4K4 binding site, and top-ranked compounds were selected as potential inhibitors (Figure 1(A)). Then, a kinase activity assay was used to evaluate the compounds. A compound with potent inhibition was identified. Next, we searched for analogues of the identified inhibitor and validated these analogues, which yielded a more potent MAP4K4 inhibitor. In vitro experiments were performed to understand the effect of the inhibitor in human pancreatic cancer cells, including a cell growth inhibition assay, cell viability assay, cell cycle analysis, and analysis of the MAP4K4 regulated JNK/c-Jun signalling pathway. Finally, an animal xenograft model planted with human pancreatic cancer cells and fed with the inhibitor was used to evaluate the effect on tumour growth (Figure 1(B)).

### Identification and validation of potential MAP4K4 inhibitors

Compounds used for virtual screening were obtained from the database: ChemDiv. The database contains more than 1,600,000 compounds. Compounds that contained a PAINS structure, had a QED score of < 0.25, or violated the Lipinski and Veber’s rule were removed. The remaining compounds were docked into the binding site of MAP4K4 and ranked based on the docking score. 18 top-ranked compounds were selected for the test by visually inspecting binding poses and compound availability (Table 1). The selected compounds were then tested for inhibitory activity at 1 μM by a ThermoFisher Scientific Z’LYTE kinase activity assay. Compound F389-0167 displayed the greatest reduction in MAP4K4 activity with an inhibition of 75%.

**Table 1.** Inhibitory percentage of potential MAP4K4 inhibitors.

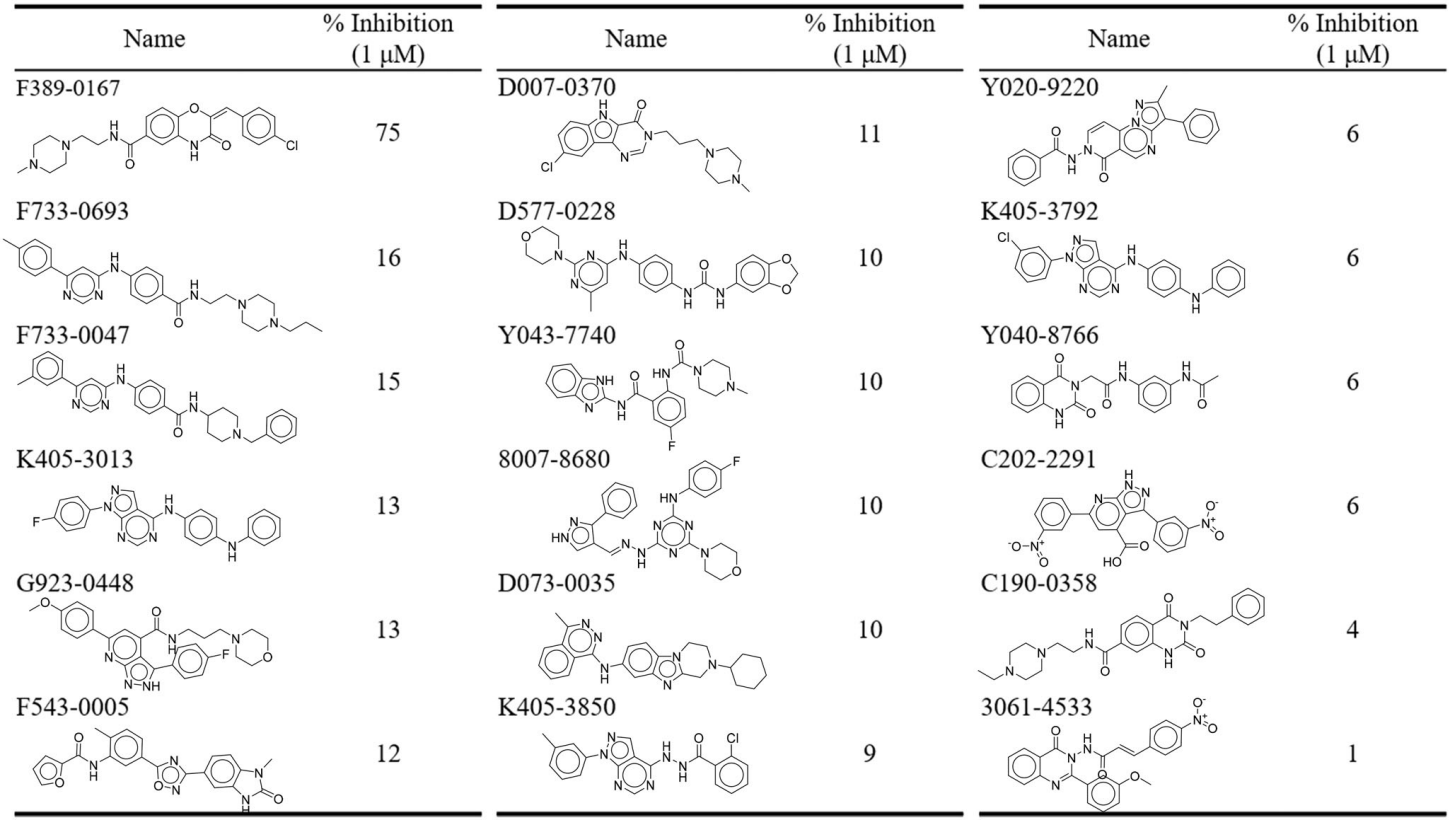					

To further study the interactions of compound F389-0167, 41 analogues were selected based on structural similarity from ChemDiv. 36 analogues containing the same core structure are presented in Table 2. The other analogues are presented separately in the supplementary materials (Supplementary Table 1). The analogues were tested for inhibitory activity at 1 μM, and F389-0746 displayed a better potency with an inhibition percentage of 87%. Five analogues (F389-0746, F389-0604, F389-0183, F389-0169, and F389-0745) exhibited inhibition percentages greater than 75%. The five analogues and the initial inhibitor, F389-0167, were selected to determine their IC_50_ values (Table 3). F389-0746 displayed the most potent inhibitory effect with an IC_50_ value of 120.7 nM, while the initial inhibitor F389-0167 had an IC_50_ value of 275.6 nM. In this study, the structure of F389-0746 was further confirmed by NMR, including ^1^H and ^13^C NMR. The NMR spectra are shown in Supplementary Figures 1 and 2.

**Figure 2.**
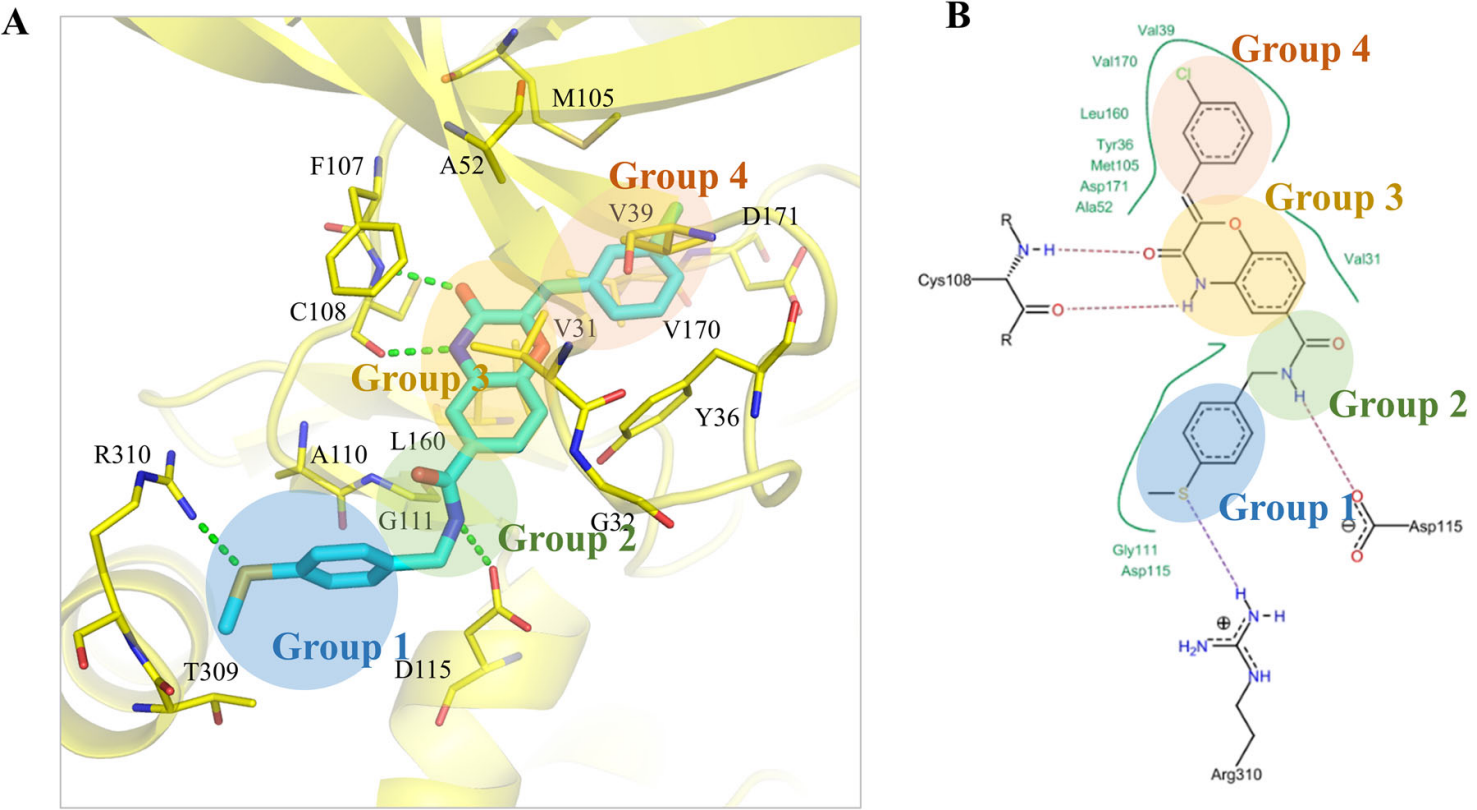
Interaction analysis of compound F389-0746. (A) Binding pose of compound F389-0746 in the MAP4K4 binding site. The dashed lines denote hydrogen bonds. The docking pose was generated by Pymol. (B) The 2D representation of compound F389-0746 docked in the MAP4K4 binding site showed hydrophobic interactions and hydrogen bonds. The dashed line denotes hydrogen bonds, and the solid line denotes hydrophobic interactions. 2D representation was created in LeadIT.

**Table 2.** Inhibitory percentage of F389-0167 analogues.

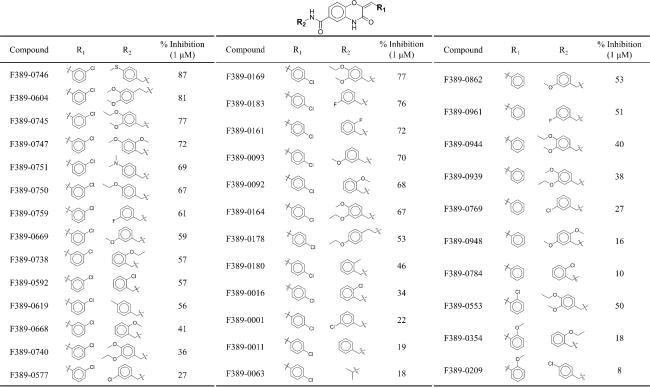											

**Table 3.** IC_50_ values of potent MAP4K4 inhibitors.

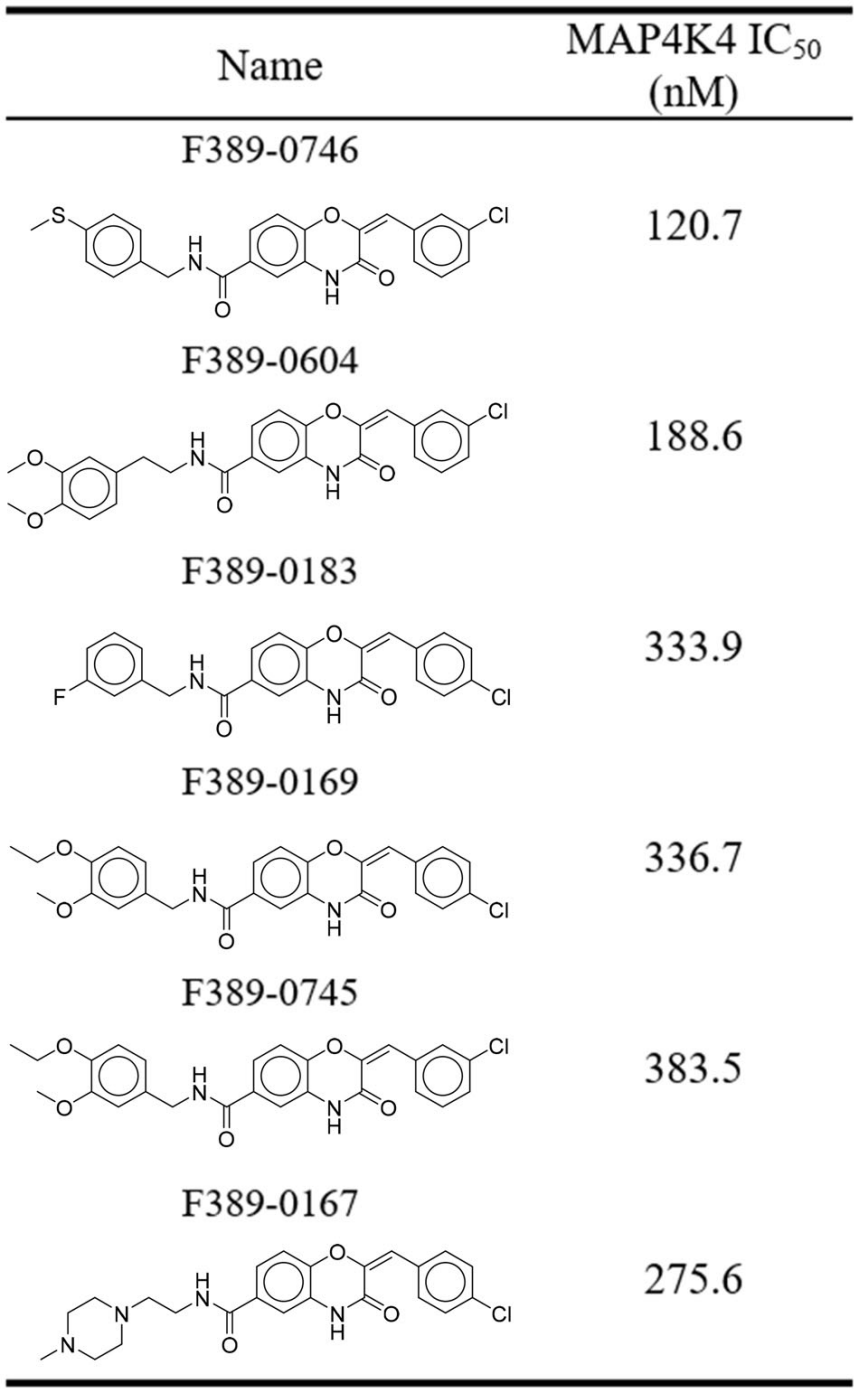	

### Interaction analysis of compound F389-0746

The binding mechanism of compound F389-0746 in the MAP4K4 binding site was demonstrated in this study (Figure 2). The structure of F389-0746 can be divided into four groups (Figure 2(A)). Group 1 contains a thiophenol moiety, and the sulphide creates hydrophobic interactions and a hydrogen bond with residue R310. Group 2 contains an amide moiety that forms a hydrogen bond with the amine of residue D115. Group 3 has a phenyl heterocyclic amide structure. The amine on the heterocycle forms a hydrogen bond with the oxygen of the hinge residue C108, while the ketone group forms another hydrogen bond with the amine of residue C108. Furthermore, the structure has stable hydrophobic interactions formed by residues V31 and L160. Group 4 includes a chlorobenzene moiety, which forms stable hydrophobic interactions with residues Y36, V39, A52, M105, L160, V170, and D171 (Figure 2(B)). In summary, these interactions could account for the potency of compound F389-0746.

### SAR analysis of F389-0746 and its analogues

**Figure 3.**
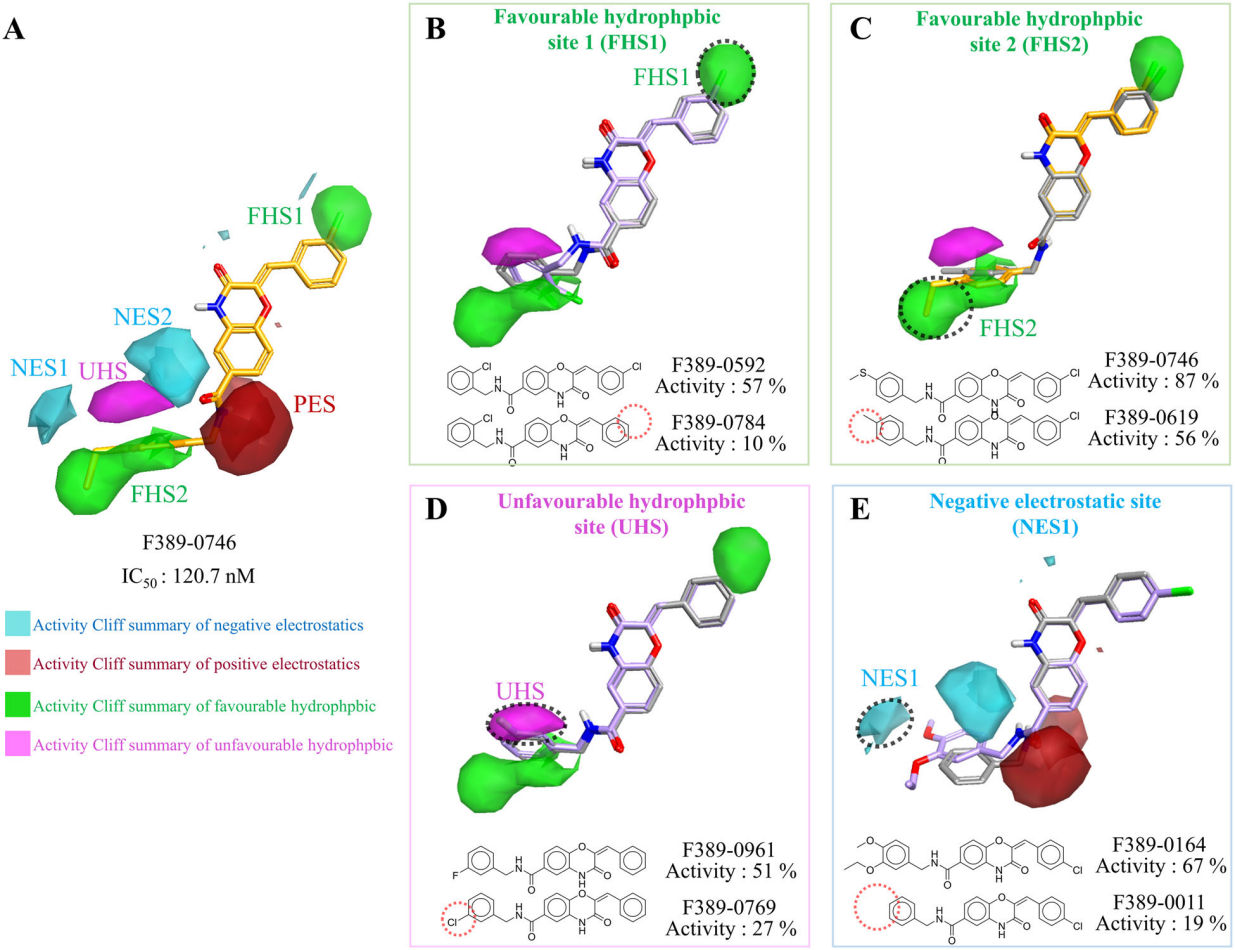
SAR analysis of F389-0746 and its analogues. (A) Favourable and unfavourable sites of interactions, including negative electrostatics, positive electrostatics , favourable hydrophobic, and unfavourable hydrophobic. (B–C) Two favourable hydrophobic sites. (D) Unfavourable hydrophobic site. (E) Negative electrostatics site. The dotted circle demonstrated the difference between the analogues and may influence the activity.

A SAR analysis was performed to further study the important interactions of the compounds using Forge software^38^. The software generated activity cliffs from the analogues with similar structures but exhibited significant differences in potency. The activity cliffs summarised favourable or unfavourable regions of hydrophobic or electrostatic interactions (Figure 3(A)). Compound F389-0746 occupies two favourable hydrophobic sites (FHS), FHS1 and FHS2, by a chloride and a thiophenol moiety, respectively. The chloride in FHS1 generates hydrophobic interactions with surrounding residues, V39 and V170, and the thiophenol moiety in FHS2 forms hydrophobic interactions with residues G111 and D115 (Figures 2 and 3(A)). An analogue, F389-0592, also contains a chloride on benzene, which extends into FHS1 (Figure 3(B)) to form the hydrophobic interactions. In contrast, F389-0784 lacks a chloride and thereby does not form the hydrophobic interactions in FHS1, resulting in a weaker inhibitory activity than F389-0592. FHS2 is occupied by the thiophenol moiety of F389-0746 (Figure 3(C)). This moiety generates hydrophobic interactions with residues G111 and D115 (Figure 2). The interactions lead to a better inhibitory activity compared to F389-0619.

In addition to the two favourable hydrophobic sites, an unfavourable hydrophobic site (UHS) adjacent to FHS1, was identified (Figure 3(D)). The analogue F389-0961 contains a fluoride in the UHS. In comparison, compound F389-0769 has a chloride in the site, leading to a lower inhibition activity than F389-0961. The activity cliffs also revealed a negative electrostatic site (NES) (Figure 3(E)). F389-0164 contains an ortho-ethoxy anisole moiety in NES1, and the moiety acts as a hydrogen bond donor and forms a hydrogen bond with residue R310, similar to the thiophenol moiety of F389-0746 in group 1 (Figure 2(B)). Compared to analogue F389-0164, analogue F389-0011 has no lone pair of electrons in the NES1 and thereby does not form a hydrogen bond with surrounding residues. As a result, a weaker inhibitory activity of F389-0011 compared to F389-0164 was observed. In summary, the SAR analysis identified favourable and unfavourable characteristics of the MAP4K4 binding site.

### Selectivity of F389-0746

**Figure 4.**
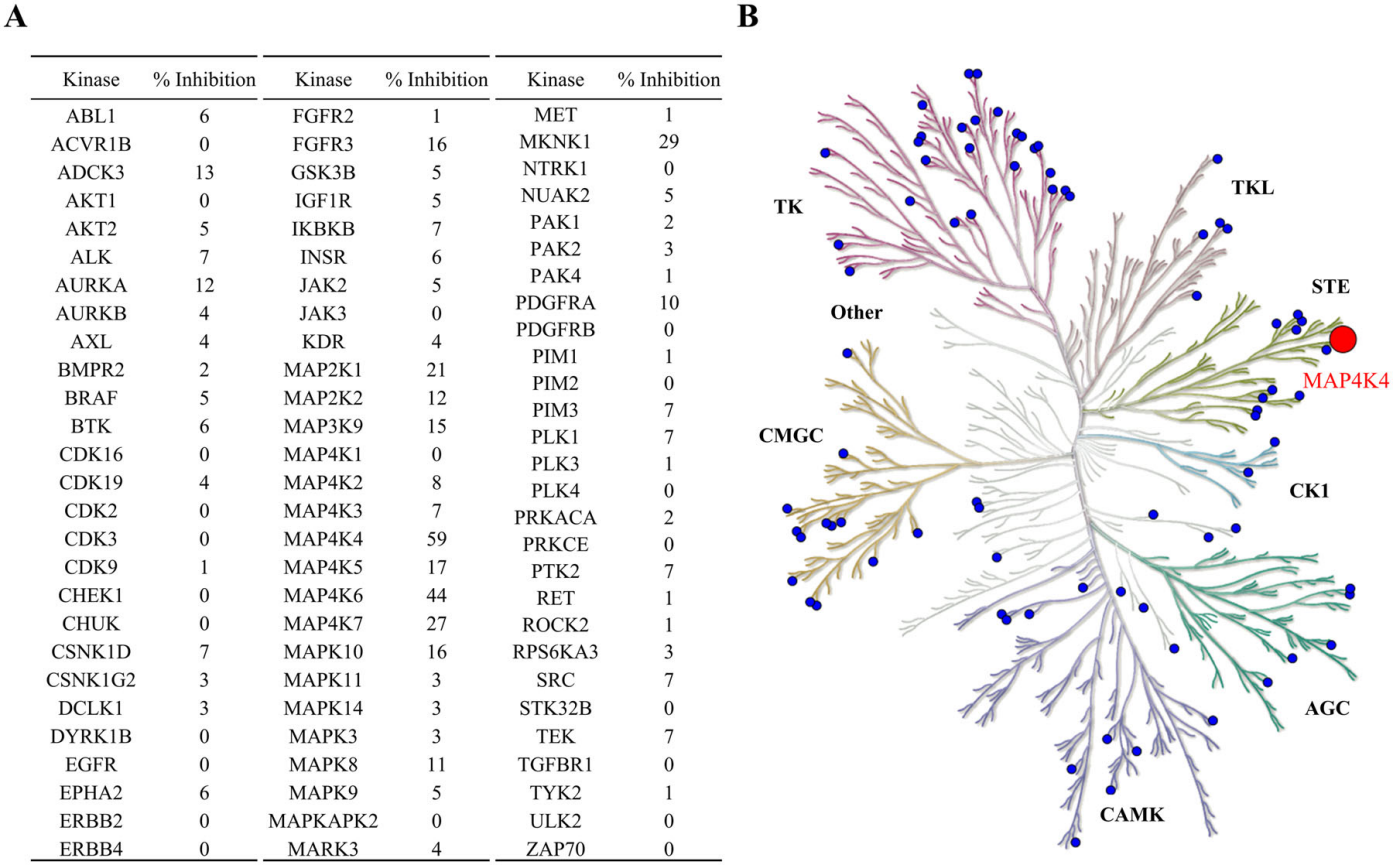
Selectivity profiling of F389-0746. (A) Inhibitory activity of F389-0746 on a panel of 81 kinases across the kinome. (B) A kinome tree composed of seven superfamilies showing the kinase inhibitory activity. The compound was tested at 150 nM. The big and the small circles represent > 50% and < 50% of inhibitions, respectively.

We further evaluated the selectivity of compound F389-0746 by kinase profiling. A panel of 81 kinases dispersed across the kinome was selected for testing. The compound was screened at 150 nM. Among the kinases in this panel, compound F389-0746 showed the best inhibitory effect with an inhibition percentage of 59% towards MAP4K4 (Figure 4(A)). The MAP4Ks family consists of 7 members: MAP4K1, MAP4K2, MAP4K3, MAP4K4, MAP4K5, MAP4K6, and MAP4K7. Of the members, compound F389-0746 showed a weaker activity inhibition for MAP4K1, MAP4K2, MAP4K3, MAP4K5, MAP4K6, and MAP4K7, with inhibition percentages of 0%, 8%, 7%, 17%, 44%, and 27%, respectively. The kinase profiling shows that F389-0746 is a selective inhibitor.

### In vitro evaluation of F389-0746 in inhibiting pancreatic cancer cells proliferation

**Figure 5.**
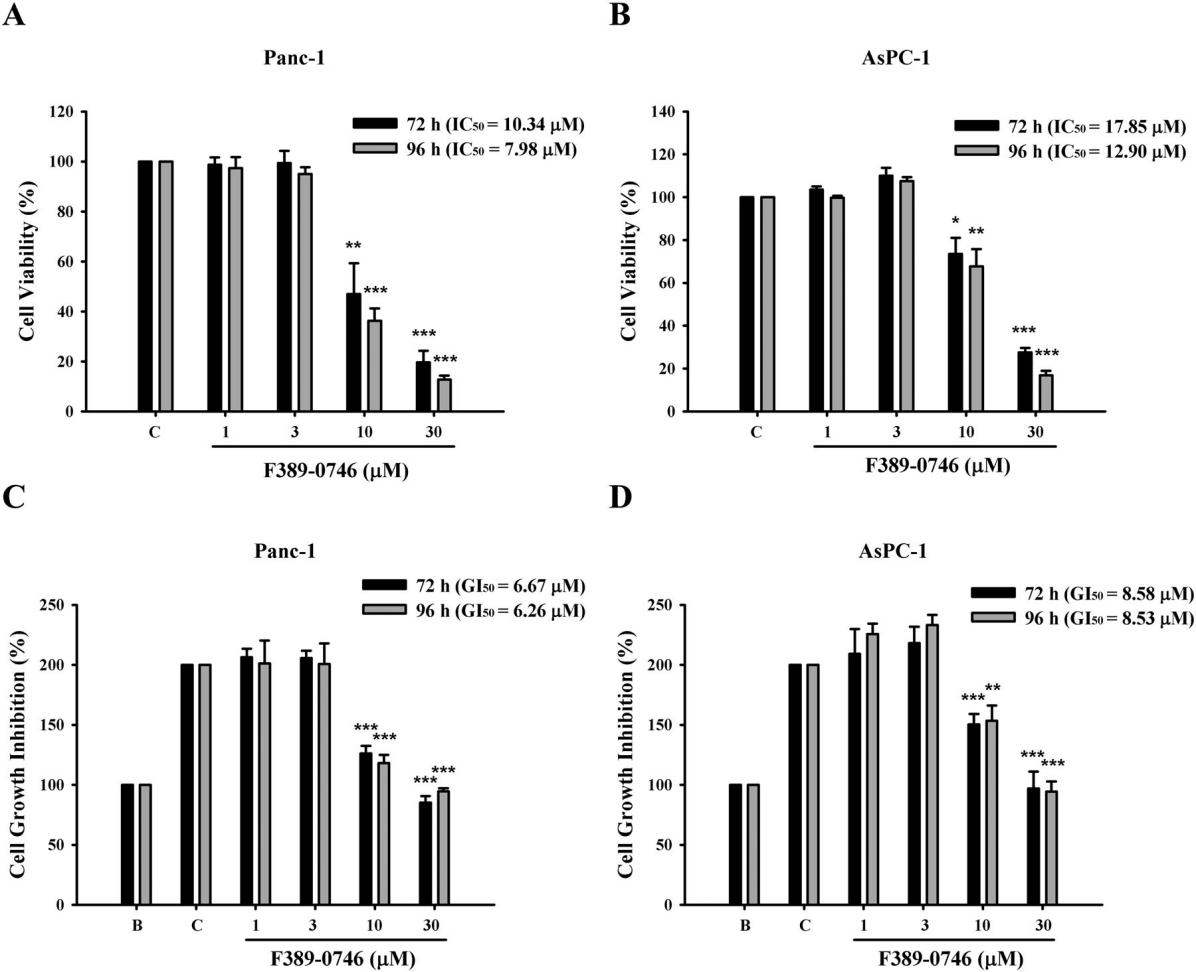
Inhibitory effects of F389-0746 on pancreatic cancer cell lines. Cell viability inhibitory effect for (A) Panc-1 and (B) AsPC-1 cells. Cell growth inhibitory effect for (C) Panc-1 and (D) AsPC-1 cells. Both cell lines were treated with 1, 3, 10, and 30 µM F389-0746 for 72 and 96 h. Results are shown as the mean ± SD from three independent experiments. Statistical significance was calculated using Student’s *t*-test. *p < 0.05, **p < 0.01, ***p < 0.001 versus the control group.

Cell viability and cell growth assays were performed to assess the anti-cancer effect of the identified MAP4K4 inhibitor, F389-0746, in human pancreatic cancer cells (Figure 5). Two human pancreatic cancer cell lines, AsPC-1 and Panc-1, were used. The cell viability and cell growth of the cell lines were inhibited when treated with F389-0746 at 10 μM for 72 and 96 h. A similar result was also observed when the compound concentration was increased to 30 μM. The IC_50_ values for 72 h were 10.34 μM and 17.85 μM in Panc-1 and AsPC-1 cells (Figure 5(A,B)), respectively. The GI_50_ values for 72 h were 6.67 μM and 8.58 μM in AsPC-1 and Panc-1 cells (Figure 5(C,D)), respectively. Moreover, for the 72- and 96-h treatments, dose-dependent inhibitory results were observed in both cell lines. Interestingly, AsPC-1 cells showed more tolerance to F389-0746 than Panc-1 cells in both treatments. These results suggest that F389-0746 can suppress cell growth and viability in both pancreatic cancer cell lines. In addition, pancreatic epithelioid carcinoma cells are more sensitive to this MAP4K4 inhibitor.

### Cell cycle distribution evaluation of F389-0746

**Figure 6.**
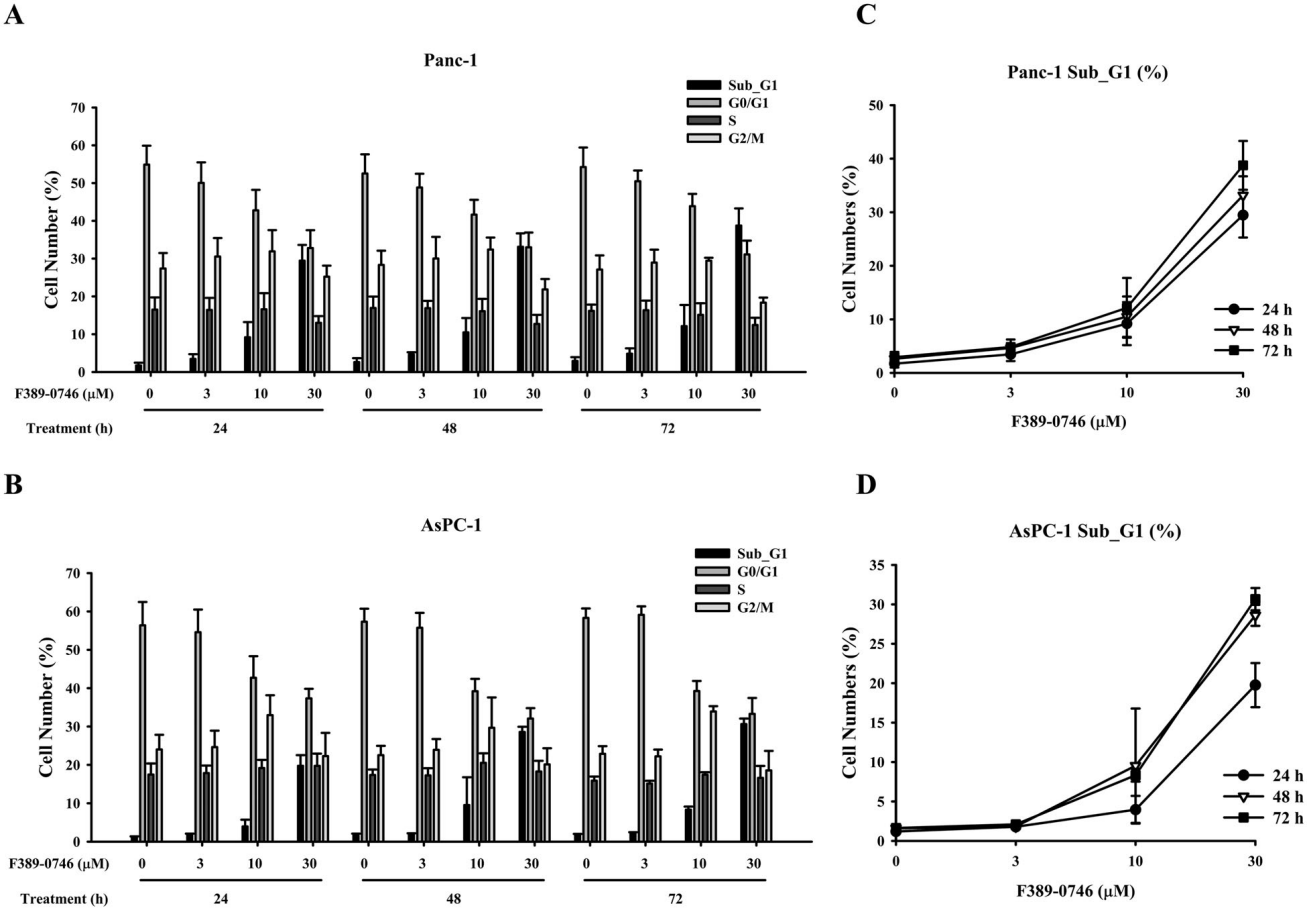
Apoptosis induction of MAP4K4 inhibitor F389-0746 in Panc-1 and AsPC-1 cells. Cell cycle distribution of (A) Panc-1 and (B) AsPC-1 cells. Percentages of SubG1 cells of (C) Panc-1 and (D) AsPC-1 cells. Both cell lines were treated with vehicles, 3, 10, and 30 μM F389-0746 for 24, 48, and 72 h. The cell cycle distribution was analysed using flow cytometry.

We next analysed the anti-proliferation effect of F389-0746 on the cell cycle distribution of AsPC-1 and Panc-1 cells. The compound was tested at 3, 10, and 30 μM for 24, 48, and 72 h using flow cytometry (Figure 6). The results of the cell cycle analysis showed that after Panc-1 and AsPC-1 cells were exposed to F389-0746 for 24 h (Figure 6(A,B)), the cell number in the subG1 group increased significantly compared with the control group, indicating that cancer cells were progressing to cell death. These phenomena were increased at 48 and 72 h in a dose-dependent manner (Figure 6(C,D)). The apoptotic cell number in the subG1 phase in Panc-1 cells was more than in AsPC-1 cells. These observations demonstrated that the MAP4K4 inhibitor, F389-0746, suppressed cell proliferation by inducing programmed cell death in pancreatic cancer cell lines.

### F389-0746 enhanced cell apoptosis by inhibiting the JNK signalling pathway

**Figure 7.**
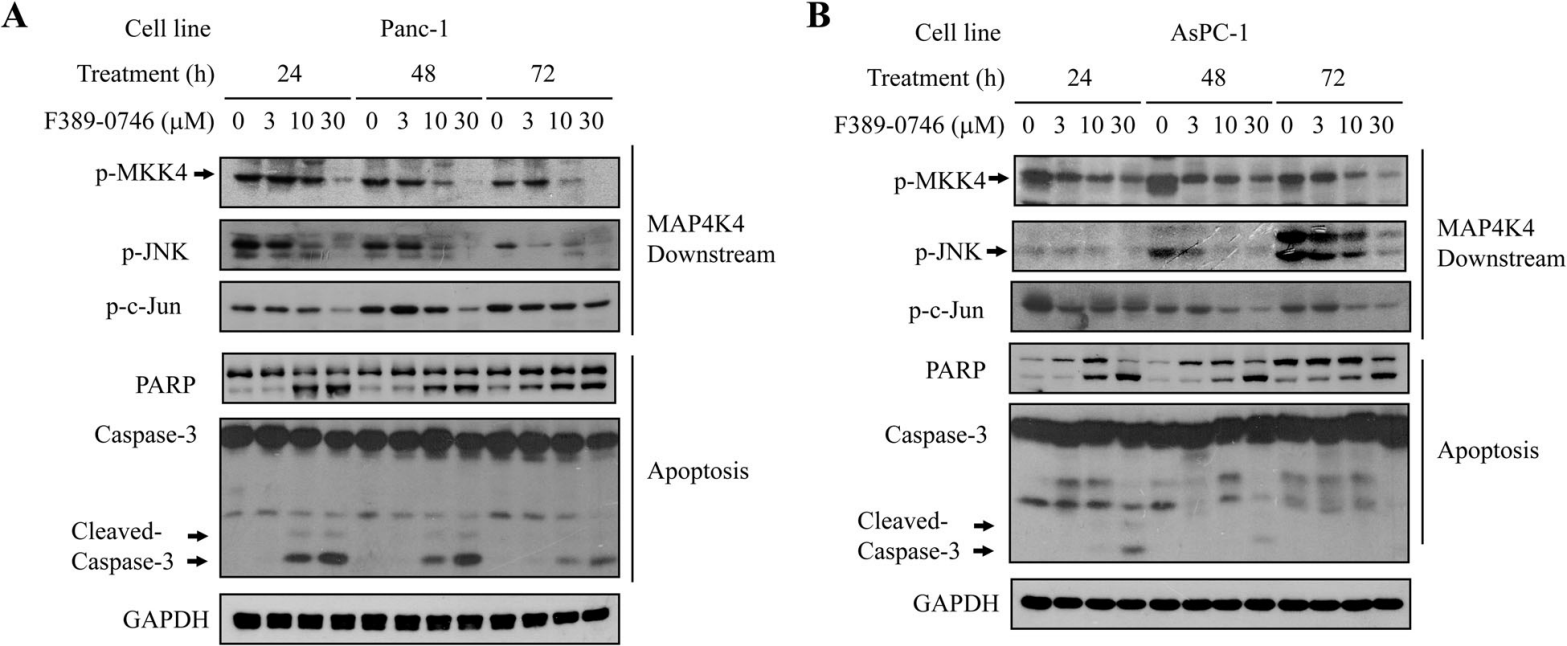
Inhibitory effects of F389-0746 on MAP4K4 downstream phosphorylation and the induction of apoptotic markers. Influence on MAP4K4 downstream phosphorylation and apoptotic markers of (A) Panc-1 and (B) AsPC-1 cells. Both cell lines were treated with F389-0746 at a concentration of 3, 10, and 30 μM for 24, 48, and 72 h.

The JNK/c-Jun pathway is the downstream signalling pathway mainly regulated by MAP4K4 and its downstream substrate MKK4 in pancreatic cancer cells^14^. Activated MAP4K4 directly phosphorylates MKK4. Phosphorylated MKK4 activates AP-1, which functions as a transcription factor for proteins associated with cell growth and proliferation. Thus, inhibiting the activity of MAP4K4 can down-regulate the JNK/c-Jun signalling pathway and suppress pancreatic cancer progression^14^. To investigate the effects of F389-0746 on the JNK/c-Jun pathway in vitro, the phosphorylation of MAP4K downstream proteins was evaluated, including MKK4, JNK, and c-Jun. The results showed that F389-0746 decreased the phosphorylation of MKK, JNK, and c-Jun in a dose-dependent manner in both cell lines (Figure 7). Furthermore, the increased cleavage of poly-ADP ribose polymerase (PARP) and caspase-3, two important apoptotic biomarkers, was found after F389-0746 treatment for 24 h (Figure 7). These results suggest that the MAP4K4 inhibitor, F389-0746, can cause pancreatic cancer cell apoptosis through the downregulation of the MKK4-JNK signalling pathway.

### In vivo evaluation of F389-0746 using a xenograft model

**Figure 8.**
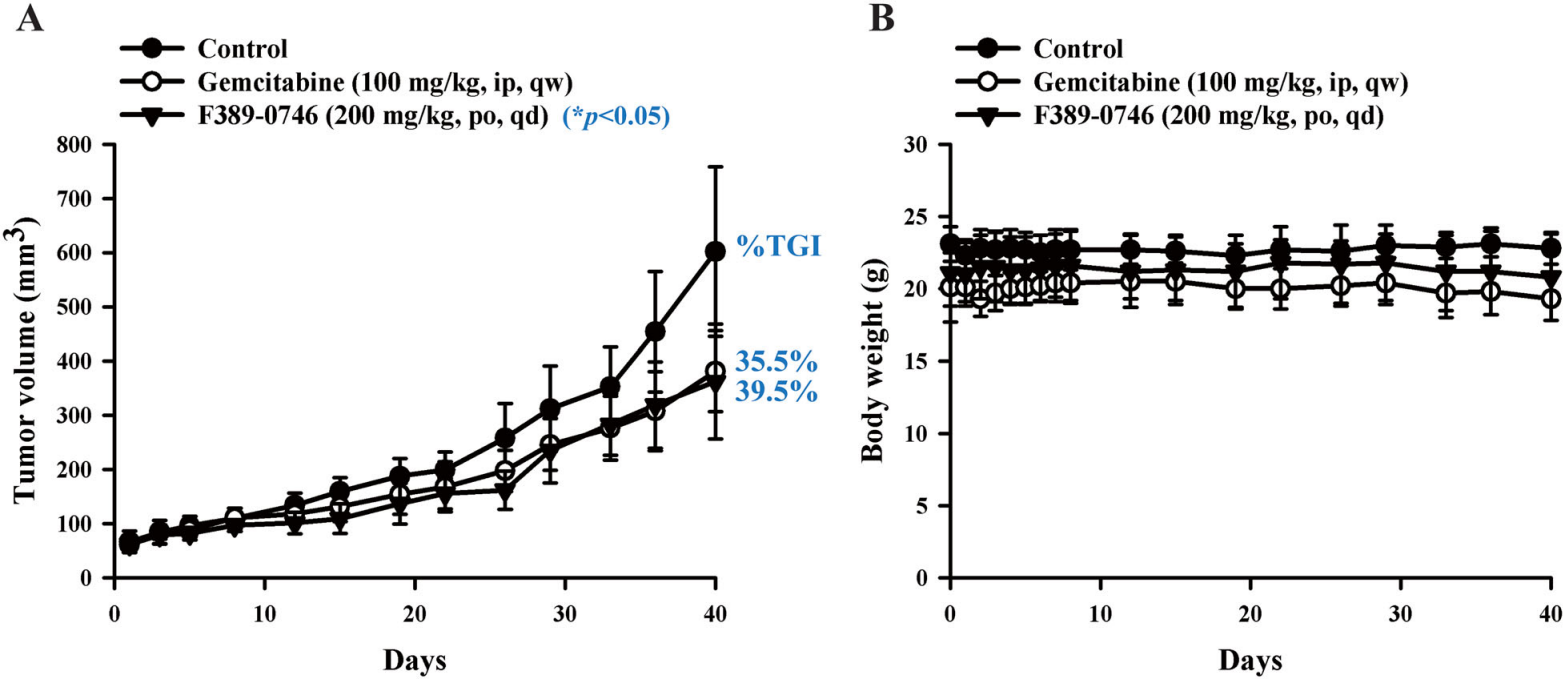
Tumour growth inhibition after F389-0746 treatment in vivo. (A) Tumour volume (B) Body weight. The control group was administered with the vehicle, the positive control group was administered with 100 mg/kg of gemcitabine, and the treatment group was administered with 200 mg/kg of F389-0746. Tumour growth inhibition (%) was calculated and compared with the control group. Results are shown as the mean ± SEM. Statistical significance was calculated using Student’s *t*-test. *p < 0.05 versus the control group.

To validate the anticancer activity of F389-0746 in vivo, we used a xenograft mouse model subcutaneously injected with Panc-1 cells (Figure 8). F389-0746 was given orally and daily in Balb/c nude mice (200 mg/kg, PO, QD). As a positive control, gemcitabine was intraperitoneally administered once a week, and tumour size was measured in all groups. Tumour size was significantly suppressed in the group treated with compound F389-0746, which showed a similar inhibitory effect to the group treated with gemcitabine (Figure 8(A)). Moreover, the group treated with F389-0746 did not display any changes in body weight (Figure 8(B)). These results indicate that the MAPK4K4 inhibitor, F389-0746, has a comparable growth inhibitory effect to gemcitabine in vivo.

## Discussion

In this study, we identified a novel MAP4K4 inhibitor using the SBVS strategy. The compound F389-0746 displayed an IC_50_ value of 120.7 nM for MAP4K4. The in vitro assays showed that the compound dose-dependently inhibited the phosphorylation of downstream proteins of MAP4K4, including MKK4, JNK, and c-Jun. The growth and proliferation of pancreatic cancer cells were also suppressed in vitro and in vivo. Along with the inhibition of MAP4K4 activity, cell apoptosis markers were induced, leading to pancreatic cancer cell death.

The high expression of MAP4K4 in pancreatic cancer was strongly correlated with poor OS and recurrence-free survival in clinical cases^15^. We compared the MAP4K4 gene expression level between normal and tumour samples from the RNAseq dataset of PAAD. The gene expression analysis of the clinical samples revealed that MAP4K4 exhibited significantly high expression (*p* < 0.001, FC = 7.96) in tumour samples compared with normal samples (Supplementary Figure 3). According to the 2-year OS analysis, the patients in the high-expression group had a significantly lower survival probability (25%) than the low-expression group (48%; log-rank *p* = 0.023; Supplementary Figure 3). These results indicate that MAP4K4 plays a key role in pancreatic cancer and may be an important target for developing new therapeutic strategies.

The MAP4K family includes seven members: MAP4K1, MAP4K2, MAP4K3, MAP4K4, MAP4K5, MAP4K6, and MAP4K7. MAP4Ks were recently identified as regulators of large tumour suppressor 1/2 (LATS1/2), a core effector of the Hippo signalling pathway^46^. This signalling pathway is responsible for controlling organ size and tissue homeostasis by manipulating cell division or cell apoptosis^47–49^. Even though many MAP4Ks were found to be overexpressed in tumour sites and play important roles in promoting cancer^50–53^, broad suppression of MAP4K functions may lead to the dysregulation of the Hippo signalling pathway and eventually contribute to tumourigenesis^54^^,^^55^. Thus, a selective MAP4K4 inhibitor would suppress cell growth and proliferation in pancreatic cancer with less dysregulation of the Hippo signalling pathway. However, developing selective MAP4K4 inhibitors is challenging due to the similar structures of all MAP4Ks^56^. Current MAP4K4 inhibitors may affect other MAP4Ks and induce unwanted side effects. For example, MAP4K4-IN-3 and PF-06260933 not only inhibit MAP4K4 but also hit MAP4K2 and MAP4K5^28^^,^^57^. In addition, compound MAP4K4-IN-3 displayed 75.2% and 63.6% inhibition activity towards MAP4K2 and MAP4K5, respectively, at a concentration of 1 μM. In this study, we identified a selective MAP4K4 inhibitor, F389-0746 (Figure 4(A)). This compound displayed the best inhibitory effect on MAP4K4 among all MAP kinase isoforms, where 59% of MAP4K4 activity was inhibited at 150 nM. Compound F389-0746 is thus less likely to cause side effects due to its selectivity.

The in vitro data showed that F389-0746 inhibited the cell growth and viability of Panc-1 and AsPC-1 cells (Figure 5). In addition, the inhibition of MAP4K4 activity induced pancreatic cancer cell apoptosis (Figure 6(C,D)). Interestingly, these results showed that F389-0746 had a better inhibition ability in Panc-1 cells than AsPC-1 cells. This phenomenon might be attributed to the different expression levels of MAP4K4 in pancreatic epithelial cells and adenocarcinoma cells. The level of MAP4K4 can vary due to the different characteristics of cells^58^. A compound could display better inhibition against cancer cells with a higher expression level of its target protein^59^^,^^60^. Previous studies indicated that MAP4K4 is expressed at higher levels in the pancreatic neoplastic epithelium compared to the pancreatic adenocarcinoma stroma^15^^,^^61^^,^^62^. Therefore, we evaluated the MAP4K4 expression level of the two pancreatic cancer cell lines in this study (Supplementary Figure 4). Compared to the pancreatic adenocarcinoma cells, AsPC-1, the pancreatic epithelial cells, Panc-1, expressed a higher level of MAP4K4 expression (Supplementary Figure 4). Because of the high abundance of MAP4K4 in pancreatic epithelial cells, inhibiting the activity of MAP4K4 could effectively suppress the growth and proliferation of the Panc-1 cell line. As a result, Panc-1 cells were more sensitive to compound F389-0746 administration with lower IC_50_ and GI_50_ values than AsPC-1 cells.

The MAP4K4 selective inhibitor, F389-0746, identified in this study showed potency (IC_50_=120.7 nM) and high selectivity. In the in vitro experiments, a relatively higher cellular IC_50_ was observed (Figure 5). Previous research suggests that this difference may be caused by low cellular permeability^63^. Compound modification by eliminating polar, reducing hydrophilic moieties, or adding lipophilic moieties could improve the compound cellular permeability and increase cellular activity^63^. For example, Lavendustin A shows an excellent inhibitory effect on tyrosine kinase activity (IC_50_=11 nM). However, in cell experiments, due to its low cellular permeability, Lavendustin A displays a poor inhibitory ability to tyrosine kinase-induced cell proliferation^63^. After attaching a methyl ester moiety to Lavendustin A, the penetration of Lavendustin A methyl ester to cells is increased, thereby inhibiting the proliferation of cells^63^. Compared with Lavendustin A, Lavendustin A methyl ester displayed an increased anti-proliferative activity IC_50_ value from over 100 μM to 0.04 μM^64^. According to the results above, structure optimisation for F389-0746 may enhance the cellular permeability of the compound and increase cellular activity.

MAP4K4 was demonstrated to promote pancreatic cancer cell growth and proliferation by activating downstream MKK4-JNK-AP-1 signalling, which enhances the translation of proliferative proteins and cytokines. Our findings suggest that the suppression of proliferation by F389-0746 was via the downregulation of these MKK4-JNK pathways (Figures 5 and 7). In addition, a similar result was demonstrated in vivo. The compound F389-0746 presented comparable tumour growth inhibition with gemcitabine (Figure 8). Furthermore, a high percentage of pancreatic cancer cells underwent apoptosis in the cell cycle analysis (Figure 6), and caspase activation, an apoptotic biomarker, was observed after treating with F389-0746 for over 24 h (Figures 6 and 7). These phenomena might be due to MAP4K4 regulating other signalling pathways to manipulate cell apoptosis, such as the Notch and NF-κB signalling pathways^10^. For example, deactivation of these signalling pathways was discovered to induce pancreatic cancer cell apoptosis^65^^,^^66^. Hence, F389-0746-induced apoptosis was possibly influenced by other signalling pathways, but further investigation is needed.

In the preclinical animal experiments, most studies used intraperitoneal (IP) injection instead of intravenous (IV) injection of gemcitabine to improve the convenience of administration^67^. Injections, whether through IV in clinical patients or IP in preclinical animal studies, display a high rate of bioavailability. However, for convenience, safety, and patient compliance, many anti-cancer drugs are expected to be taken orally^63^. Many FDA-approved small-molecule kinase inhibitors are effective when taken orally^63^. Orally administrating F389-0746 in vivo and comparing it to gemcitabine, which is injected into the murine models, would give indications of its effectiveness as an oral therapeutic. According to our in vivo results, orally administered F389-0746 displays a similar tumour growth inhibitory effect to IP-injected gemcitabine (Figure 8). The above results suggest that F389-0746 has the potential to be further optimised as an oral drug.

In summary, MAP4K4 plays an important role in pancreatic cancer. Through regulating the downstream JNK signalling pathway, MAP4K4 promotes cancer cell proliferation, growth, and metastasis. In this study, we used an SBVS approach to identify MAP4K4 inhibitors and successfully found a novel MAP4K4 inhibitor with an IC_50_ value of 275.6 nM. An analogue, F389-0746, was then identified to exhibit better MAP4K4 inhibitory activity with an IC_50_ value of 120.7 nM. Analysing the analogues revealed favourable and unfavourable interactions in the MAP4K4 binding site. Additionally, F389-0746 showed high selectivity towards MAP4K4, which is less likely to cause side effects. When validated in vitro, F389-0746 inhibited the phosphorylation of MAP4K4 downstream proteins, including MKK4, JNK, and c-Jun, in the JNK signalling pathway. Furthermore, cellular processes regulated by the JNK signalling pathway were suppressed, including cancer cell growth and proliferation. Pancreatic cancer cells undergoing apoptosis were also observed due to the lack of survival or proliferation-related proteins. Compound F389-0746 also displayed anticancer activity in a xenograft pancreatic model in vivo. Animals treated with compound F389-0746 displayed comparable tumour growth inhibition to those treated with gemcitabine. These results suggest that the MAP4K4 inhibitor, F389-0746, has the potential to be developed as a novel targeted therapy drug for pancreatic cancer treatment.

## Supplementary Material

Supplemental Material
