# Supplementary material for: In silico identification and biological evaluation of a selective MAP4K4 inhibitor against pancreatic cancer
Source: J Enzyme Inhib Med Chem. 2023 Jan 22;38(1):2166039. doi: 10.1080/14756366.2023.2166039 (PMC9873280; doi:10.1080/14756366.2023.2166039)
Supplement: Supplemental Material [file IENZ_A_2166039_SM5289.pdf]

Supplementary Tables and Figures

In silico identification and biological evaluation of a selective MAP4K4 inhibitor against pancreatic cancer

Chao-Di Chang[a,1], Min-Wu Chao[b,c,1], Hsueh-Yun Lee[a,d,j], Yi-Ting Liu[d], Huang-Ju Tu[e], Ssu-Ting Lien[e], Tony Eight Lin[e,f], Tzu-Ying Sung[g], Shih-Chung Yen[h], Sing-Han Huang[i], Kai-Cheng Hsu[a,e,f,j,k,l,*], Shiow-Lin Pan[a,e,f,j,k,*]

[a]Ph.D. Program in Drug Discovery and Development Industry, College of Pharmacy, Taipei Medical University, Taipei, Taiwan
[b]School of Medicine, College of Medicine, National Sun Yat-sen University, Kaohsiung, Taiwan
[c]Institute of Biopharmaceutical Sciences, College of Medicine, National Sun Yat-sen University, Kaohsiung, Taiwan
[d]School of Pharmacy, College of Pharmacy, Taipei Medical University, Taipei, Taiwan;
[e]Graduate Institute of Cancer Biology and Drug Discovery, College of Medical Science and Technology, Taipei Medical University, Taipei, Taiwan
[f]Ph.D. Program for Cancer Molecular Biology and Drug Discovery, College of Medical Science and Technology, Taipei Medical University, Taipei, Taiwan
[g]Biomedical Translation Research Center, Academia Sinica, Taipei, Taiwan
[h] Warshel Institute for Computational Biology, The Chinese University of Hong Kong (Shenzhen), Shenzhen, Guangdong, People's Republic of China
[i]Graphen Inc., New York, NY 10110, USA
[j]TMU Research Center of Cancer Translational Medicine, Taipei Medical University, Taipei, Taiwan
[k]TMU Research Center for Drug Discovery, Taipei Medical University, Taipei, Taiwan
[l]Cancer Center, Wan Fang Hospital, Taipei Medical University, Taipei, Taiwan

*Corresponding authors
E-mail: piki@tmu.edu.tw (K.C. Hsu), slpan@tmu.edu.tw (S.L. Pan)
[1]These authors contributed equally to this work.

Information Classification: General

Supplementary Table 1. Inhibitory percentage of F389-0167 analogues.

Compound	Structure	% Inhibition (1 μM)
K788-4810	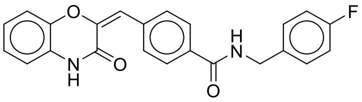	28
K788-8871	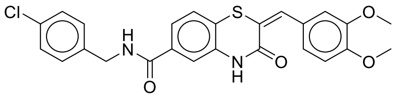	26
K788-6734	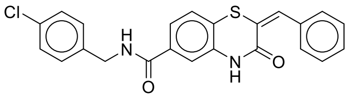	24
K788-4405	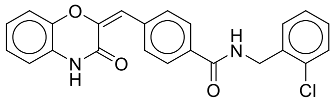	24
K788-9488	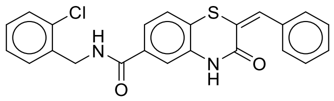	4

Information Classification: General

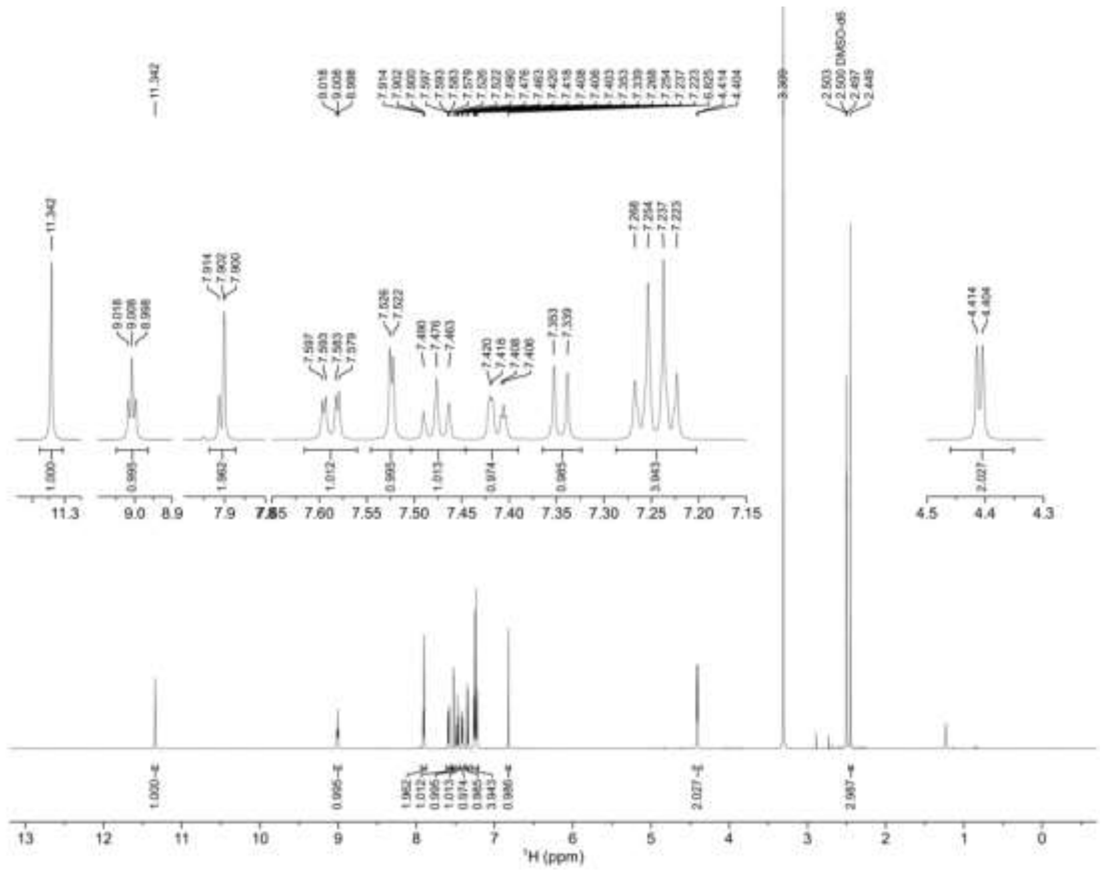

Supplementary Figure 1. ^{1}H NMR Spectrum for F389-0746.

Information Classification: General

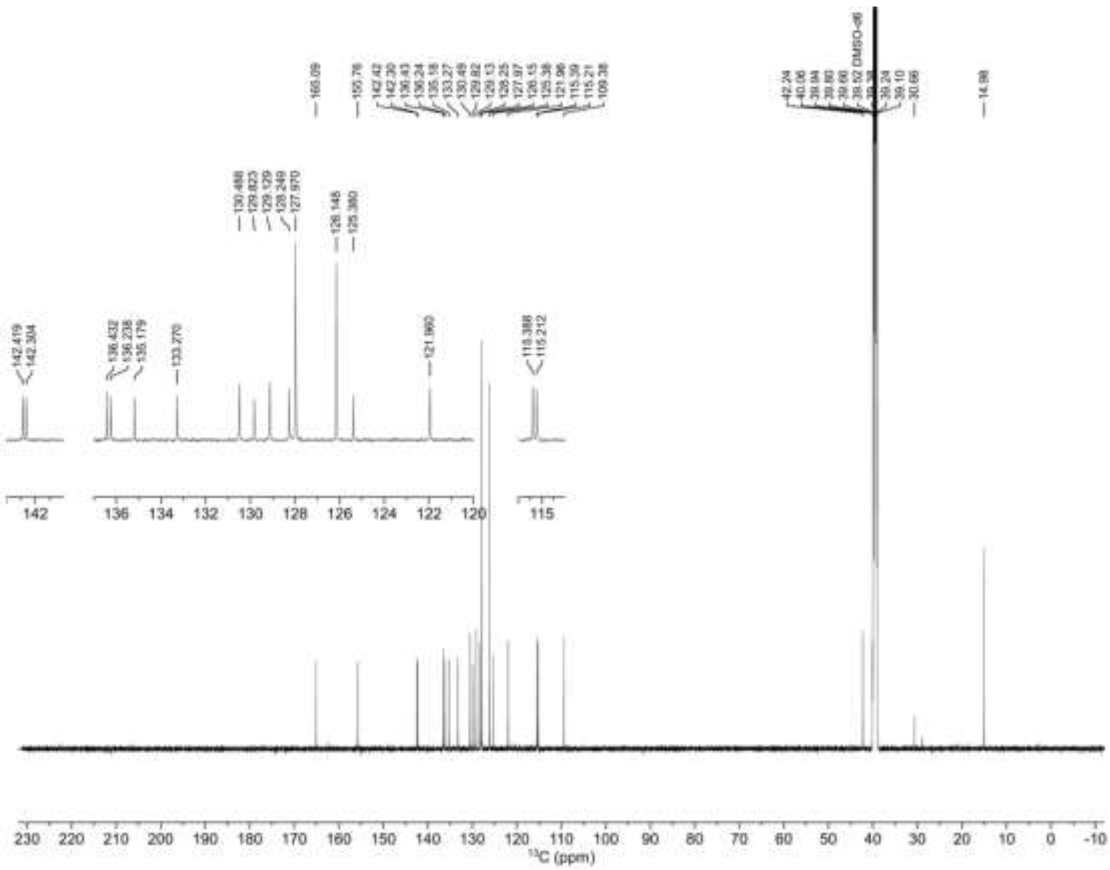

Supplementary Figure 2. ^{13}C NMR Spectrum for F389-0746.

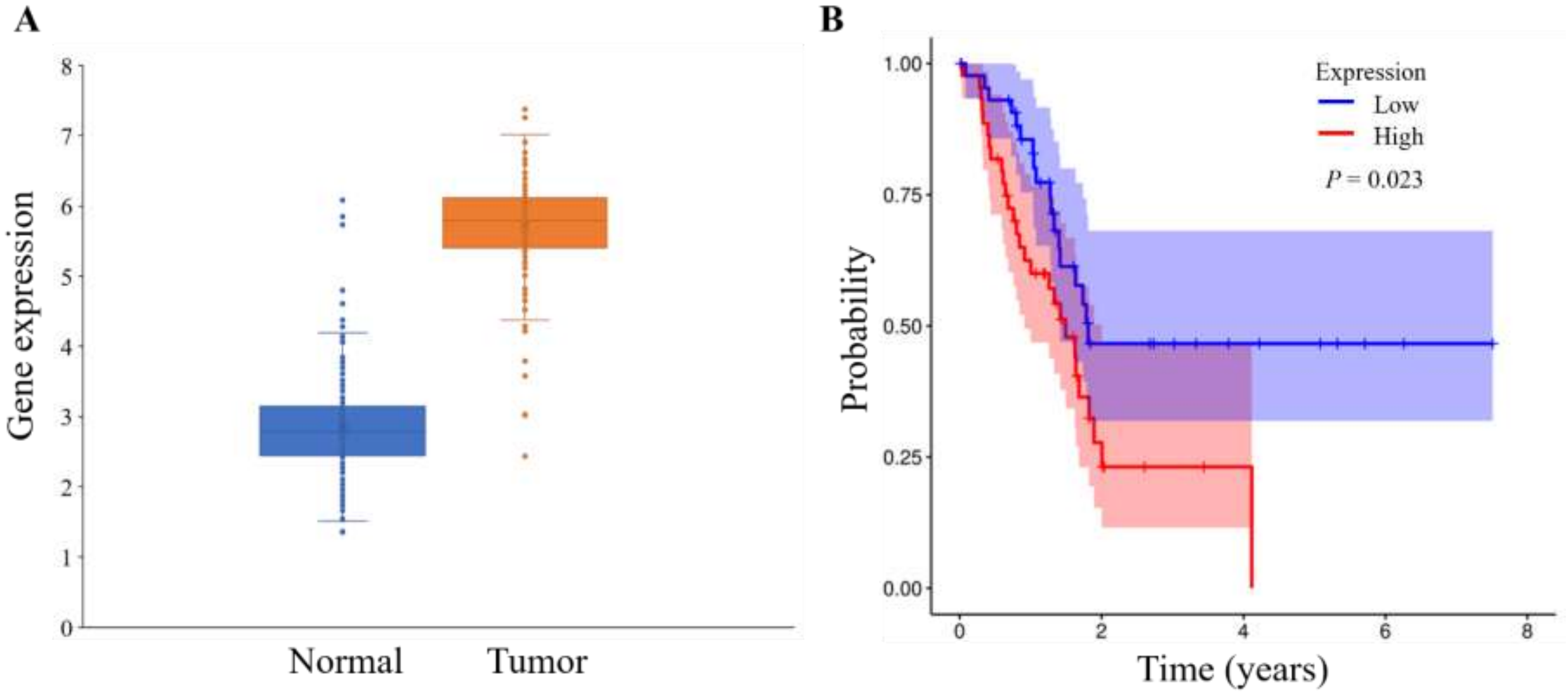

Supplementary Figure 3. Differential expression and survival analyses between high and low MAP4K4 groups. (A) Gene expression level of MAP4K4 of normal and tumor pancreatic samples. (B) Overall survival analysis of pancreatic cancer patients with high or low MAP4K4 expression levels.

Information Classification: General

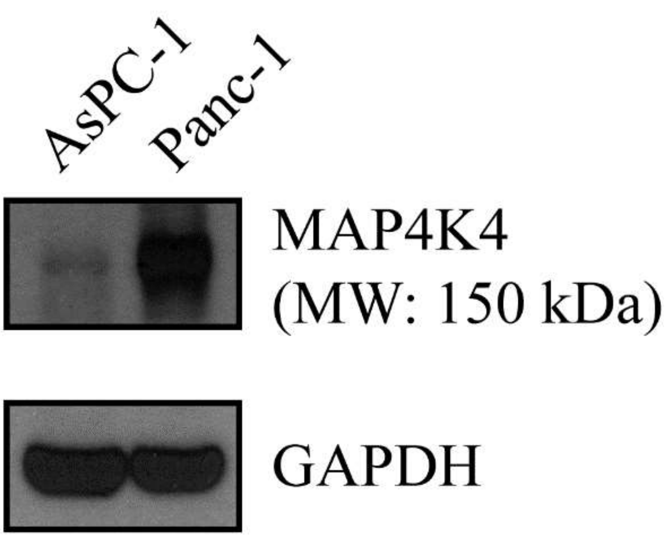

Supplementary Figure 4. Expression level of MAP4K4 in pancreatic cancer cell lines, AsPC-1 and Panc-1.

Information Classification: General

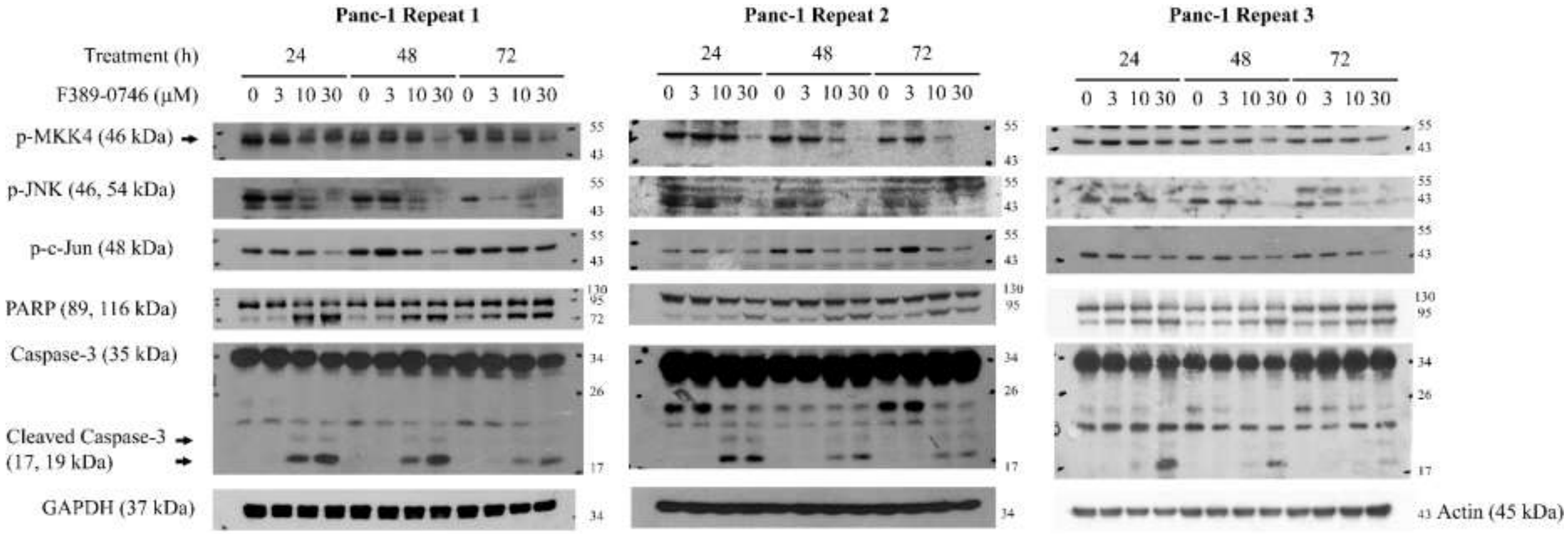

Supplementary Figure 5. Three independent repeats of Panc-1 cells treated with F389-0746

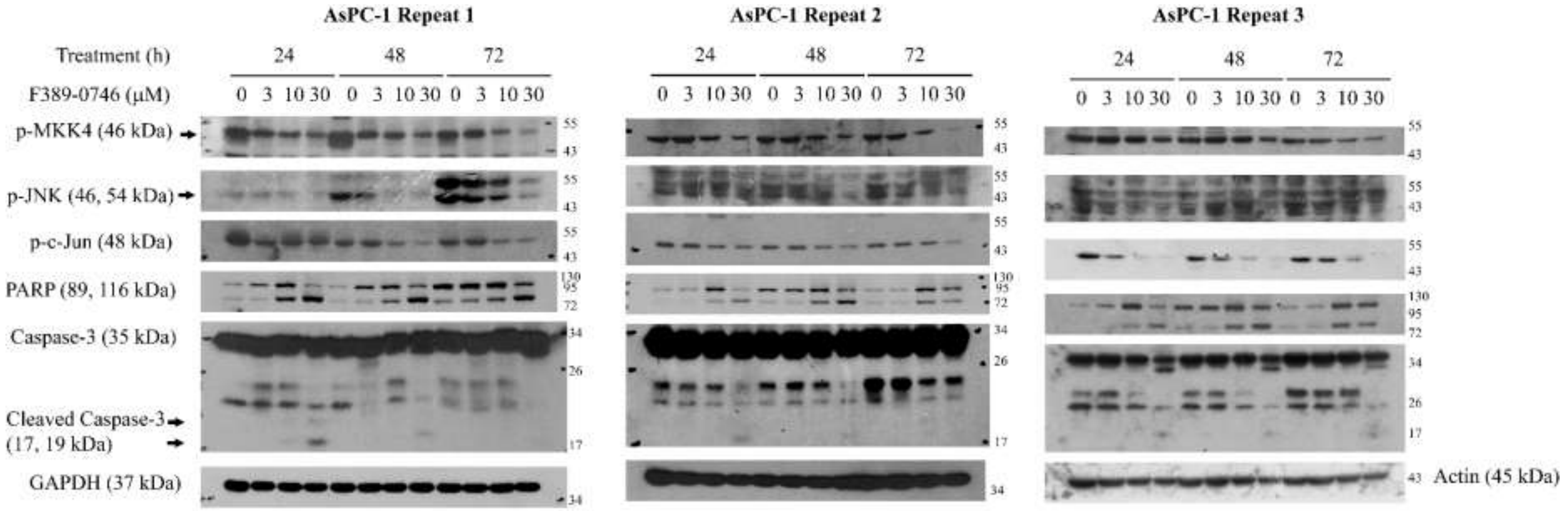

Supplementary Figure 6. Three independent repeats of AsPC-1 cells treated with F389-0746
